# The antipsychotic agent trifluoperazine hydrochloride suppresses triple-negative breast cancer tumor growth and brain metastasis by inducing G0/G1 arrest and apoptosis

**DOI:** 10.1038/s41419-018-1046-3

**Published:** 2018-09-26

**Authors:** Zhanzhan Feng, Yong Xia, Tiantao Gao, Fuyan Xu, Qian Lei, Cuiting Peng, Yufei Yang, Qiang Xue, Xi Hu, Qianqian Wang, Ranran Wang, Zhiqiang Ran, Zhilin Zeng, Nan Yang, Zixin Xie, Luoting Yu

**Affiliations:** 1 Lab of Medicinal Chemistry, State Key Laboratory of Biotherapy and Cancer Center, West China Hospital, Sichuan University and Collaborative Innovation Center for Biotherapy, 610041 Chengdu, China; 2 School of Chemical Engineering, Sichuan University, 610041 Chengdu, China; 3 Sichuan Yuanda Shuyang Pharmaceutical Co., Ltd., 610041 Chengdu, China; 4 West China School of Pharmacy, Sichuan University, 610041 Chengdu, China

## Abstract

Women with aggressive triple-negative breast cancer (TNBC) are at high risk of brain metastasis, which has no effective therapeutic option partially due to the poor penetration of drugs across the blood−brain barrier. Trifluoperazine (TFP) is an approved antipsychotic drug with good bioavailability in brain and had shown anticancer effect in several types of cancer. It drives us to investigate its activities to suppress TNBC, especially the brain metastasis. In this study, we chose three TNBC cell lines MDA-MB-468, MDA-MB-231, and 4T1 to assess its anticancer activities along with the possible mechanisms. In vitro, it induced G0/G1 cell cycle arrest via decreasing the expression of both cyclinD1/CDK4 and cyclinE/CDK2, and stimulated mitochondria-mediated apoptosis. In vivo, TFP suppressed the growth of subcutaneous xenograft tumor and brain metastasis without causing detectable side effects. Importantly, it prolonged the survival of mice bearing brain metastasis. Immunohistochemical analysis of Ki67 and cleaved caspase-3 indicated TFP could suppress the growth and induce apoptosis of cancer cells in vivo. Taken together, TFP might be a potential available drug for treating TNBC with brain metastasis, which urgently needs novel treatment options.

## Introduction

Breast cancer is the most common cancer and the second leading cause of malignancy death among women in America and its incidence is increasing globally^1^. About 246,660 new cases of invasive breast cancer were expected to be diagnosed and nearly 40,450 women died of this disease in the United States in 2016^2^. Triple-negative breast cancer (TNBC) is the most aggressive subtype of breast cancer^3,4^. Although lots of time and funds had been put into the research of TNBC and some new targets emerged, metastatic TNBC is still difficult to treat for lack of effective specific target, resulting in extremely poor survival^5^. The current conventional therapeutic strategies for treating TBNC failed to achieve a satisfactory result^1,6^. Brain metastasis is the end stage of the devastating disease in breast cancer progression^7^. Currently there is no effective treatment option available including radiotherapy, which could only prolong patients’ lives by a few months^8,9^. Brain metastasis is a great challenge in this new era of personalized targeted cancer therapies. Therefore, it urgently needs great effort to discover effective therapeutic strategies and actionable molecular targets to cure TNBC patients with brain metastasis.

Dysregulation of cell cycle is a hallmark of cancer. Cell cycle is an accurate process responsible for the proper division of one cell into two daughter cells^10–12^. The genetic control of cell division is dysfunctional in cancer, leading to an unrestricted cell proliferation^13^. Disrupting cell cycle can inhibit proliferation and induce apoptosis of tumor, in favor of the therapy of cancer^10,14^.

Apoptosis is a programmed process of cell death, which plays an important role in eliminating unwanted cells in damaged multicellular organism. It also works in a variety of biological process, including cell differentiation and proliferation^15,16^. Dysregulation of apoptosis leads to numerous diseases including cancer and is another hallmark of cancer^12^. Therefore, compounds that could block cell cycle and induce apoptosis might be effective therapeutic agents for treating TNBC.

Nowadays, the development of anticancer drugs is more difficult than before. It is accompanied with some big challenges caused by increasing failure rates, high cost, poor bioavailability that cannot be solved, unwanted safety and limited efficacy in clinical trials. Exploring approved noncancer drugs for their anticancer activities could decrease the failure of development and save time and money^17^. Some studies showed schizophrenic patients using neuroleptic agents have less risk of cancer^18,19^. Trifluoperazine (TFP) is a phenothiazine derivative commonly used as antipsychotic drug. Limited studies have reported that TFP has anticancer efficacies^20^. However, there were few reports about the investigation of TFP in treating TNBC. Antischizophrenic agent like TFP could easily penetrate the blood−brain barrier (BBB) to achieve a high concentration in brain, leading us to investigate its activities to treat TNBC and the brain metastasis.

The aim of our study was to obtain some insight into the activities of TFP against TNBC in vitro and in vivo along with the underlying mechanisms. We found that TFP could induce G0/G1 cell cycle arrest of TNBC cells via decreasing the expression level of cyclinD1/CDK4 and cyclinE/CDK2 complexes. It could also induce apoptosis of the cancer cells via the reactive oxygen species (ROS)–mitochondrial apoptotic pathway. Moreover, TFP could suppress TNBC cells migration and invasion. Importantly, TFP inhibited the growth of established subcutaneous xenograft tumor and the brain metastasis of TNBC without causing obvious side effects.

To the best of our knowledge, there was no report about TFP’s potential application in treating established TNBC brain metastases. Given it’s an approved drug, TFP could be rapidly advanced into clinical trial. Our results suggested that TFP may be a potential antitumor candidate and its further investigation is warranted.

## Results

### TFP inhibited TNBC cells proliferation

**Fig. 1:**
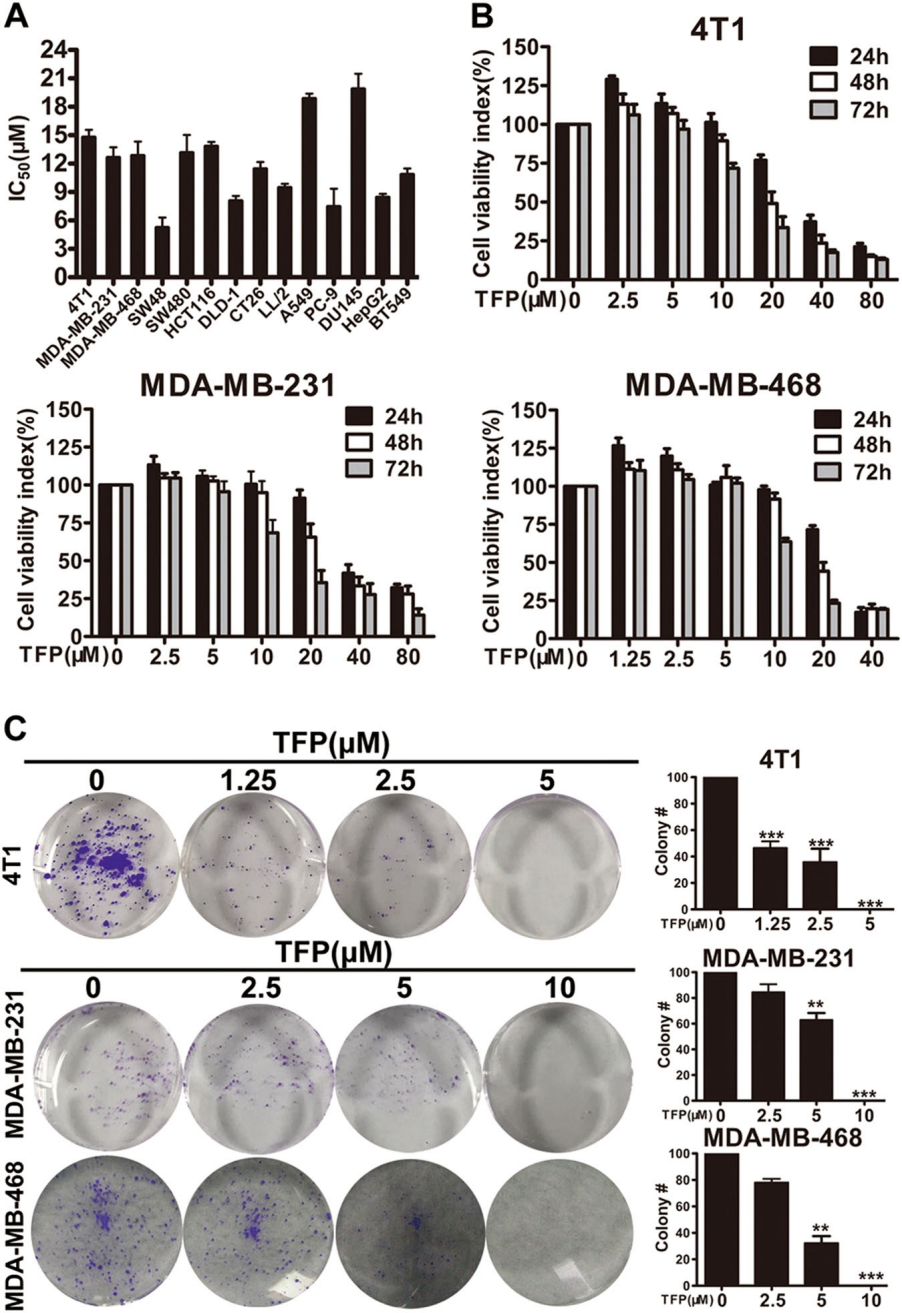
TFP inhibited TNBC cells proliferation. **a** Effects of TFP on the viabilities of cancer cells after 72 h of treatment, represented as the half maximal inhibitory concentration (IC_50_ values, μM). HepG2, hepatoma carcinoma cells; SW48, SW480, HCT116, DLD-1, CT26, colorectal cancer cells; A549, PC-9, non-small-cell lung carcinoma cells; LL/2, mouse Lewis lung carcinoma cells; DU145, prostate cancer cells; and others are TNBC cells. **b** 4T1, MDA-MB-231, and MDA-MB-468 cells were treated with various concentrations of TFP for 24, 48 and 72 h, respectively. Cell viability was evaluated by MTT assay. Values represent mean ± SD (*n* = 3, in triplicate). **c** The effect of TFP on colony formation in 4T1, MDA-MB-231, and MDA-MB-468 cell lines for 7–10 days. The statistic results of colony formation assays are presented as survival colonies. The quantification is shown in right panels. Data were expressed as mean ± SD. from three experiments (**P < 0.01; ***P < 0.001); the treatment group and the control group were compared by *t* test

To evaluate the effects of TFP on cell viability, several cell lines were exposed to TFP. The results indicated that TFP could reduce their survival with IC_50_ values less than 20 μM (Fig. 1a). We are interested in exploring new drugs for TNBC. Therefore human TNBC cell lines MDA-MB-231, MDA-MB-468, and mouse TNBC cell line 4T1 were chosen for further studies.

We evaluated the effects of TFP on cell viability using the three TNBC cell lines, respectively. The viabilities were reduced with increasing TFP concentration and longer treatment time (Fig. 1b). These results indicated that TFP could suppress TNBC cells viability in a time- and concentration-dependent manner. To further elucidate the effects of TFP on breast cancer cell proliferation, we performed colony formation assay. As shown in Fig. 1c, the number of colonies in the three cell lines was reduced in a concentration-dependent manner after TFP treatment. Taken together, these results suggested that TFP had strong cytotoxic and cytostatic effects on TNBC cells.

### TFP induced G0/G1 phase arrest of TNBC cells

**Fig. 2:**
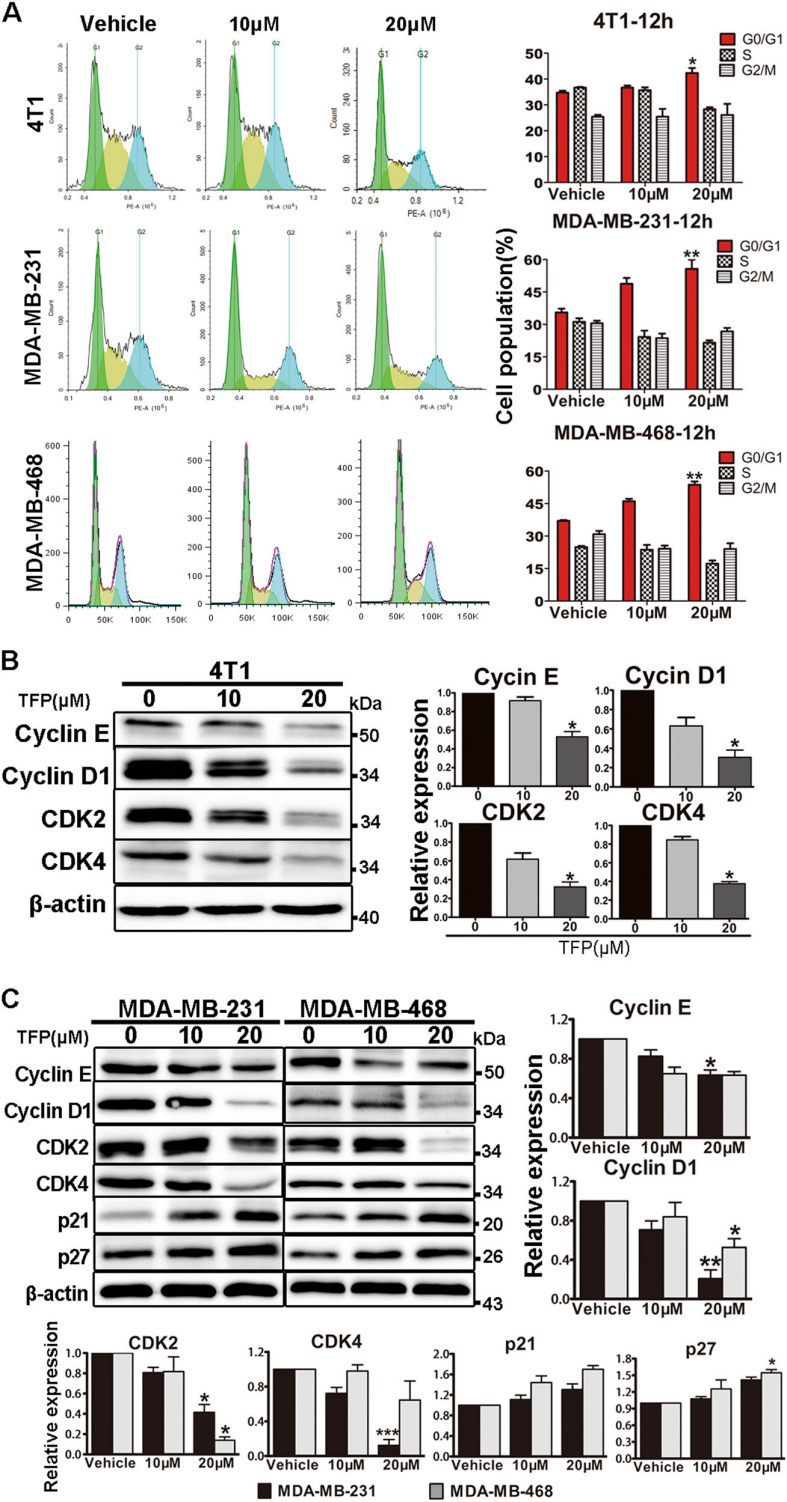
TFP induced G0/G1 arrest in TNBC cells. **a** 4T1, MDA-MB-231, and MDA-MB-468 cells were treated with indicated concentration of TFP for 12 h, respectively. The distribution of cell cycle was analyzed by flow cytometry. Data shown were representative of three independent experiments. The quantification is shown in right panels. Data were expressed as mean ± SD from three experiments (*P<0.05; **P < 0.01); the treatment group and the control group were compared by *t* test. **b**, **c** The effects of TFP on the expression of cyclin E, cyclin D1, CDK2, CDK4, p21, and p27 were determined by western blotting. β-actin was used as the loading control. The quantified values were shown (*P < 0.05; **P<0.01; ***P < 0.001); the treatment group and the control group were compared by *t* test

To further illustrate the molecular mechanisms underlying the anti-TNBC effect of TFP, we studied its effect on cell cycle distribution by flow cytometry (FCM) analysis in 4T1, MDA-MB-231, and MDA-MB-468 cells. Notably, as shown in Fig. 2a, G0/G1 phase arrest was induced in a concentration-dependent manner after incubation with TFP for 12 h. The percentage of 4T1 cells in G0/G1 phase was increased from 33.5% in the vehicle group to 42.7% in the group of 20 μM TFP treatment for 12 h and 34.1−53.6% for 24 h (Supplementary Fig. 2A). Similar results were observed in MDA-MB-231 and MDA-MB-468 cells (Fig. 2a and Supplementary Fig. 2A). These data reflected that G0/G1 phase arrest might be associated with the effects of TFP against TNBC.

Cell cycle is precisely regulated by various proteins including cyclins and cyclin-dependent kinases (CDKs). Cyclin D, cyclin E and their relevant CDKs are crucial regulators in G1 phase^21–23^. Therefore, we evaluated the expression of proteins involved in cell cycle by western blot. As shown in Fig. 2b, c, the expression of cyclin E, CDK2, cyclin D1, CDK4 decreased after treatment with TFP for 72 h in 4T1, MDA-MB-231, and MDA-MB-468 cell lines. Next, we evaluated the expression of p27 and p21. Both of them belong to Cip/Kip cell cycle inhibitory protein (CKI) family, and the p21 is also the downstream signal molecule of p53^13^. As shown in Fig. 2c, there were increasing expression trends of p27 and p21 both in MDA-MB-231 and MDA-MB-468 cells. However, similar trend was not observed in 4T1 cells (data not shown).

### TFP induced apoptosis of TNBC cells

**Fig. 3:**
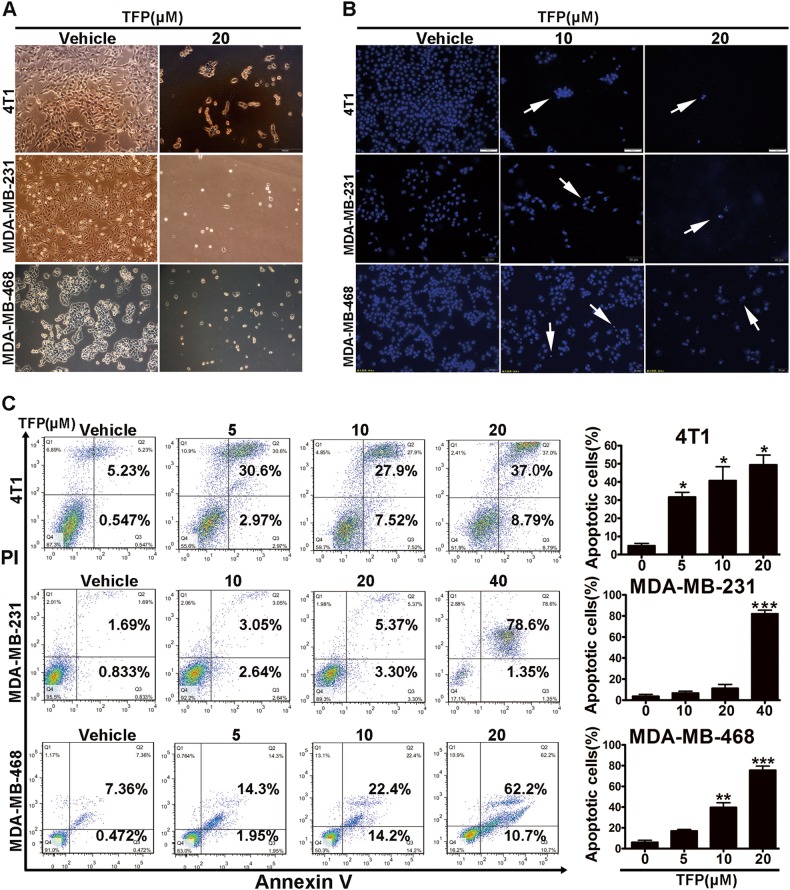
TFP induced apoptosis of TNBC cells. **a** The cell morphology of 4T1, MDA-MB-231, and MDA-MB-468 cells after treatment with TFP for 48 h. Scale bars represent 100 μm. **b** Nuclear alterations of 4T1, MDA-MB-231, and MDA-MB-468 cells after TFP treatment for 48 h. Cells were stained with Hoechst 33342 (10 μg/mL) and visualized by fluorescence microscopy. Scale bars represent 50 μm. **c** Flow cytometric analysis of TNBC cells apoptosis using the Annexin V/PI dual-labeling technique after TFP treatment for 48 h. The quantified values were shown in right panels (*P < 0.05; **P<0.01; ***P < 0.001); the treatment group and the control group were compared by *t* test

The morphological changes of the nucleus were observed in 4T1, MDA-MB-231, and MDA-MB-468 cells after TFP treatment. As shown in Fig. 3a, cell shrinkage was induced after 20 μM TFP treatment for 48 h, which indicated the induction of apoptosis. To confirm the induced apoptosis, Hoechst 33342 staining was performed. Bright-blue fluorescent condensed nuclei and formation of apoptotic bodies were observed in 4T1, MDA-MB-231, and MDA-MB-468 cells after TFP treatment for 48 h (Fig. 3b). These findings confirmed the apoptosis.

Next, we quantified the effects of TFP on apoptosis with FCM after Annexin V/PI staining. As shown in Fig. 3c, both early apoptotic fraction (Annexin V positive and PI negative) and late apoptotic fraction (Annexin V and PI positive) were increased in a concentration-dependent manner in TNBC cells after TFP treatment. Quantitatively, the apoptosis rate increased from 2.5% in the vehicle group to 5.7% and 8.6% when treated with 10 and 20 μM TFP for 48 h in MDA-MB-231 cells, respectively, consistent with the previous findings^24^. Those findings suggested TFP could induce apoptosis in TNBC cells in a concentration-dependent manner. Activation of caspase-3, which was confirmed by the decrease of procaspase-3 and the increased expression of cleaved caspase-3 after TFP treatment in TNBC cell lines, confirmed the apoptosis (Supplementary Fig. 4A).

### TFP induced apoptosis via the mitochondria-mediated apoptotic pathway

**Fig. 4:**
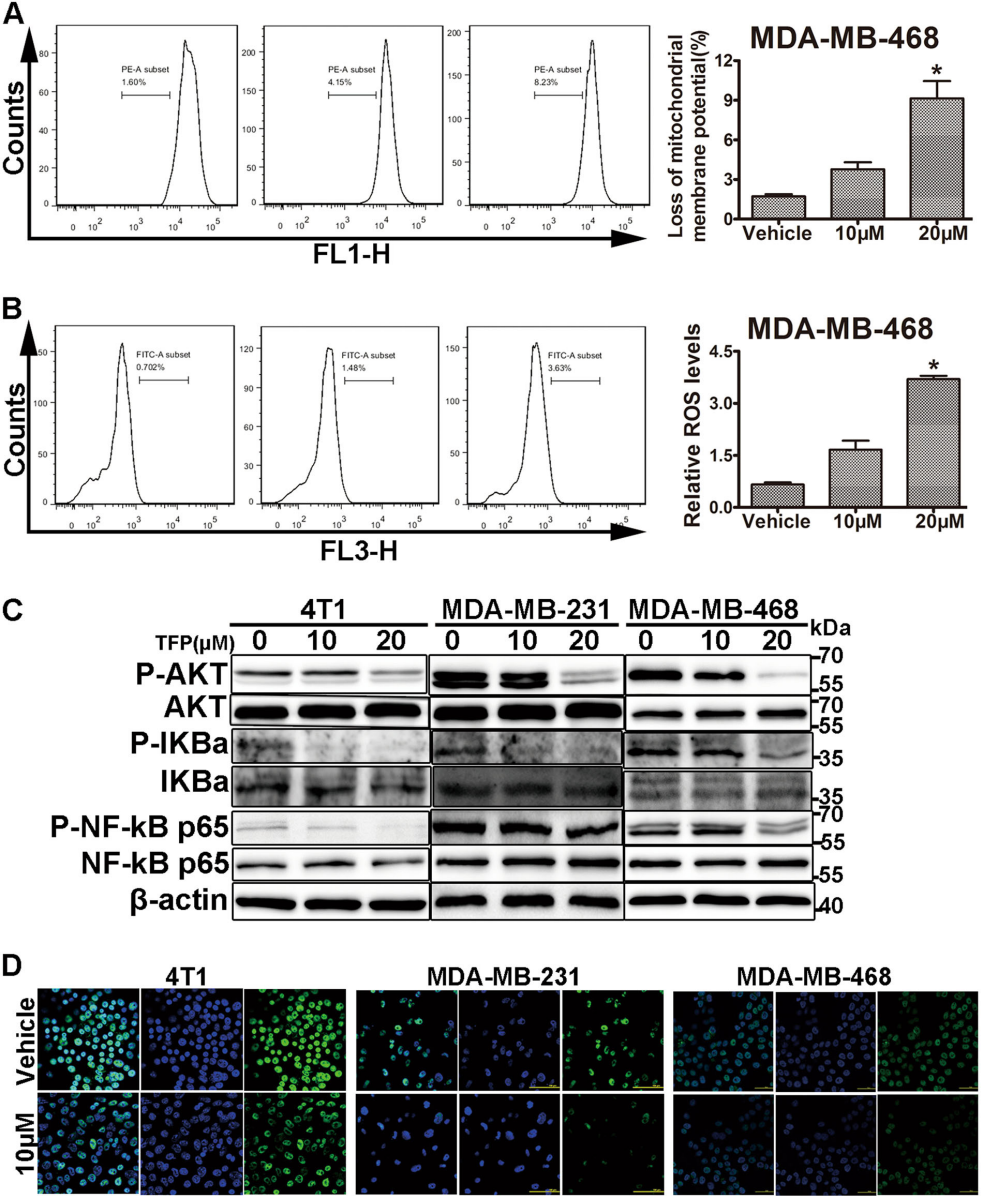
TFP induced mitochondria-mediated apoptosis of TNBC cells. **a** TFP treatment decreased mitochondrial membrane potential (ΔΨm) in MDA-MB-468 cells. Cells were treated with various concentrations of TFP for 24 h and then stained with Rh123 to measure the change of ΔΨm by flow cytometry. Quantified values are shown on the right (*P < 0.05); the treatment group and the control group were compared by *t* test. **b** TFP increased ROS level in MDA-MB-468 cells. After treatment with various concentrations of TFP for 12 h, MDA-MB-468 cells were incubated with DCFH-DA and then ROS levels were measured by DCF fluorescence with flow cytometry. Quantified values were shown on the right (*P < 0.05). **c** The expression of related proteins was determined by western blotting. 4T1, MDA-MB-231, and MDA-MB-468 cells were treated with TFP for 72 h and the expression of P-AKT, AKT, P-IKBa, IKBa, P-NF-kB p65, and NF-kB p65 were detected. β-actin served as internal control. **d** TFP inhibited the NF-kB p65 nuclear translocation. The intracellular localization of NF-kB p65 was determined by immunofluorescence. Pictures were taken with a laser-scanning confocal microscopy (Nikon). Scale bars represent 50 μm

There are two major pathways of apoptosis. One is the death-receptor pathway, the other is the mitochondrial pathway. Both of them are regulated by the caspase cascade^25^. To further investigate which pathway involved in TFP-induced apoptosis, we analyzed the expression of Bcl-2 and Bax, both of which regulate the mitochondrial apoptosis, in the three TNBC cell lines treated with TFP. As shown in Supplementary Fig. 4A and B, the expression of antiapoptotic Bcl-2 remarkably decreased, while the proapoptotic Bax increased in a concentration-dependent manner, and an obvious increase of Bax/Bcl-2 expression ratio was observed (Supplementary Fig. 4C). Disruption of mitochondrial membrane permeability and loss of mitochondrial membrane potential (ΔΨm) are important events in the intrinsic apoptotic pathway. To further elucidate TFP-induced apoptosis, changes in ΔΨm were detected with FCM. As shown in Fig. 4a, there was a significant loss of ΔΨm in MDA-MB-468 cells after 24 h of TFP treatment. Similar results were observed in 4T1 and MDA-MB-231 cells (Supplementary Fig. 4D and 4F). These results indicated that the mitochondrial apoptotic pathway might involve in TFP-induced apoptosis.

ROS is mainly produced by mitochondria and excess ROS could lead to cell death^26^. Increasing ROS level in cancer cells causes intrinsic apoptosis^27–29^. As shown in Fig. 4b, Supplementary Fig. 4E and G, the ROS level in the three TNBC cells increased in a concentration-dependent manner after treatment with TFP for 12 h.

Many signaling pathways involved in tumor cell proliferation and apoptosis, such as PI3K-AKT-mTOR^30,31^. Abnormal activation of AKT is associated with increased cell growth, cell proliferation, metastasis, and angiogenesis. And hyperactivation of AKT had been reported in numerous cancers^30^. We investigated the effects of TFP on the expression of those proteins in TNBC cells. As shown in Fig. 4c, the expression of phosphorylated AKT was decreased without affecting its total expression level. It was reported that IKK/NF-kB signaling pathways are aberrantly activated in TNBC^24^, and AKT could regulate the activity of IKK directly or indirectly, resulting in the activation of NF-kB along with its nuclear translocation and NF-kB-dependent genes transcription, including Bcl-2 and cyclin D1, and so on, that promote cell survival^32^. Thus, we investigated the effects of TFP on the expression of related proteins involved in NF-kB signaling pathway. As shown in Fig. 4c, both phosphorylated IKBa and phosphorylated NF-kB p65 were decreased without affecting its total expression level. Furthermore, we determined the intracellular localization of p65 by immunofluorescence using a polyclonal antibody against NF-kB p65, as shown in Fig. 4d. TFP decreased the nuclear translocation of NF-kB p65. Taken the data together, TFP might have induced TNBC cells apoptosis via Akt/NF-kB signaling pathway.

### TFP inhibited subcutaneous tumor growth in TNBC xenograft models

**Fig. 5:**
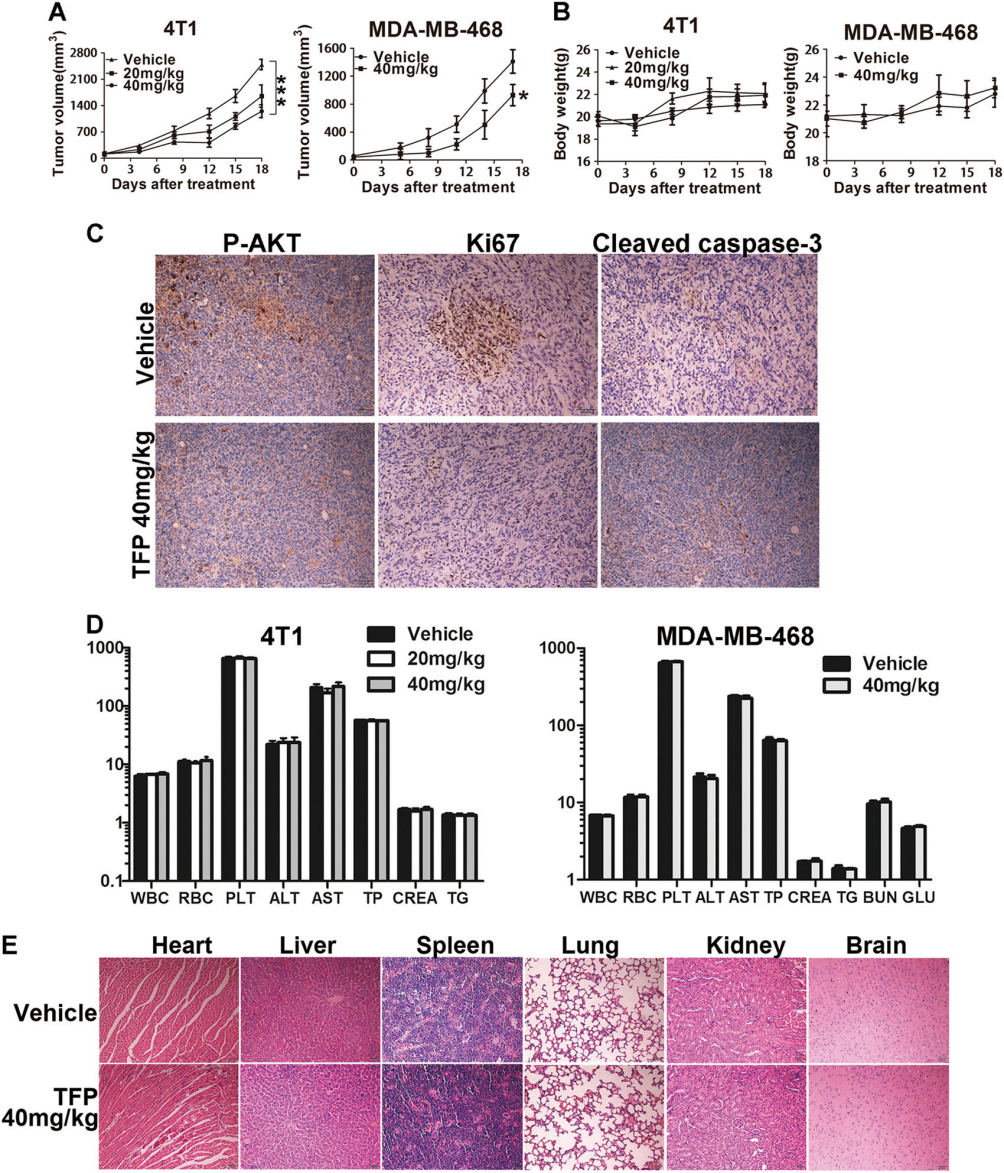
TFP’s inhibitory effects on the growth of TNBC in subcutaneous tumor model and safety profile. **a** Tumor size changes of 4T1-bearing and MDA-MB-468-bearing mice during TFP treatment. Tumor volume and body weight of the mice in each group were measured every 3 days and presented as mean ± SD (*n* = 7). ANOVA was used for statistical analysis in 4T1 subcutaneous tumor model and *t* test in MDA-MB-468 subcutaneous tumor model, *P < 0.05; ***P < 0.001. **b** Body weight changes of mice in each group. There were no significant differences between the groups. **c** Tumor tissues from the MDA-MB-468 tumor-bearing mice were immunohistochemically analyzed with Ki67, P-AKT, cleaved caspase-3. Scale bars represent 50 μm. **d** TFP treatment did not cause significant changes in blood routine analysis. Units of the parameters are as follows. WBC, PLT, 10^9^/L; RBC, 10^12^/L; TP, g/L; ALT, AST, CREA, μM; TG, mM; BUN, GLU, mM. **e** TFP treatment did not cause obvious pathologic changes in major organs of the mice. Heart, liver, spleen, lung, kidney, and brain were from the mice bearing MDA-MB-468 xenografts tumor. Images shown are representatives from each group. Scale bars represent 50 μm

To assess the potential anti-TNBC effects of TFP in vivo, 4T1 and MDA-MB-468 cells were injected subcutaneously into 6–8-week-old female BALB/c mice and NOD/SCID mice, respectively. The mice were administered daily by intraperitoneal injection (i.p.) with 20 and 40 mg/kg TFP and survived for 18 days. Vehicle-treated mice rapidly developed visible tumors, and dramatic tumor growth was observed throughout the study (Fig. 5a). In contrast, treatment with TFP attenuated the growth of tumors. These data clearly demonstrated the anti-breast cancer activity of TFP in vivo.

Then we explored the molecular mechanisms underlying the antibreast cancer effects of TFP in vivo via immunohistochemical analysis of tumor tissue. TFP treatment indeed decreased cancer cell proliferation and increased apoptosis in the tumor sections as shown by Ki67 and cleaved caspase-3 staining. Besides, the number of P-AKT-positive cells in the TFP treatment group was less compared with the vehicle group (Fig. 5c). These data indicated that reducing proliferation and inducing apoptosis also contributed to TFP’s anticancer effects in vivo and probably through AKT signaling pathways.

Besides, the TFP-treated mice in subcutaneous xenograft tumor models did not show any side effects, such as diarrhea and weight loss (Fig. 5b). As shown in Fig. 5d, there were no significant changes in hematological and serum biochemical parameters between TFP-treated mice and vehicle-treated mice. H&E staining of major organs from the mice that were treated with TFP showed no signs of pathological changes (Fig. 5e and Supplementary Fig. 5B).

### TFP inhibited breast cancer cell migration and invasion in vitro

**Fig. 6:**
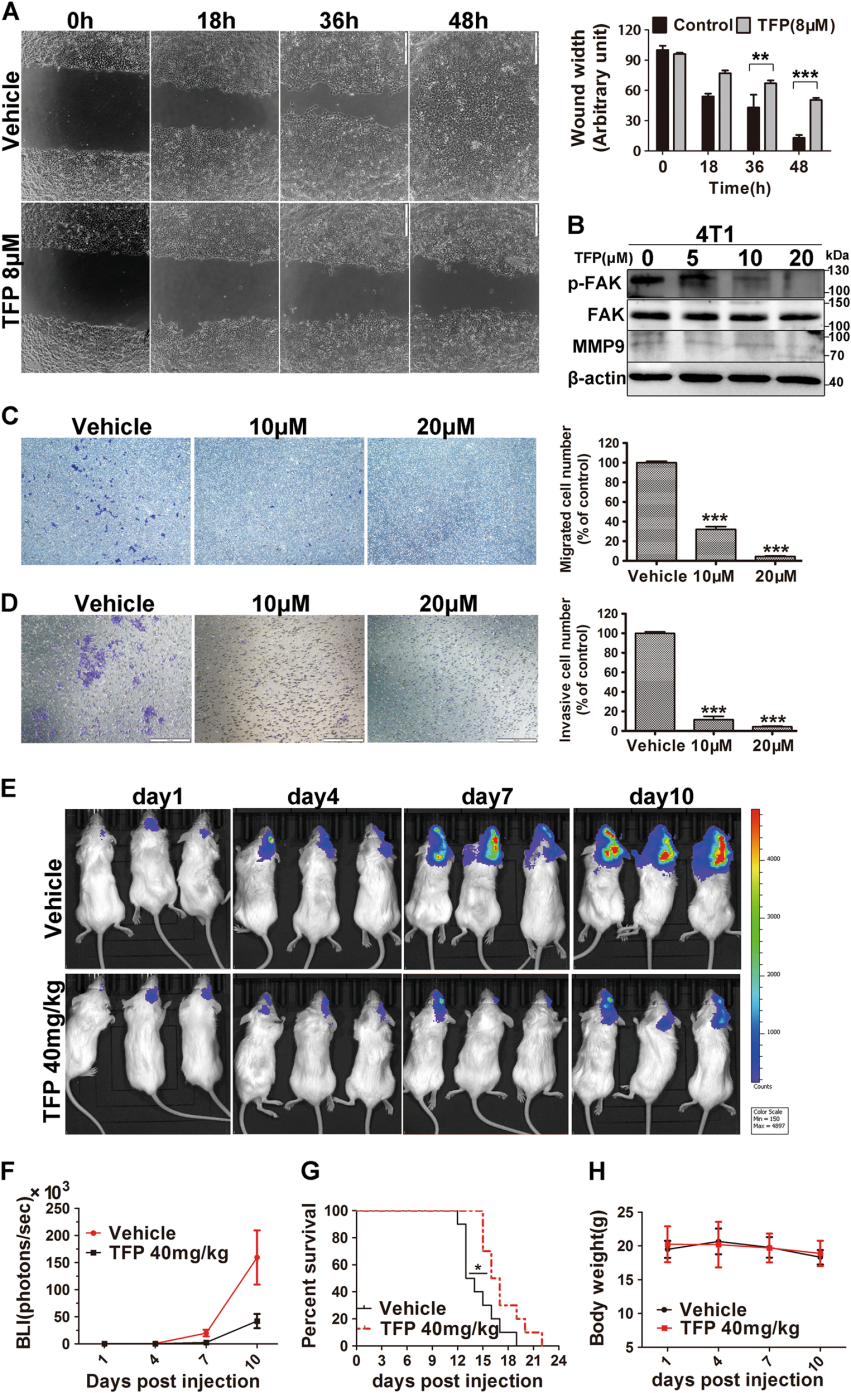
TFP’s inhibitory effects on brain metastasis of TNBC in intracarotid model. **a** TFP inhibited wound healing of 4T1 cells. Quantified values were shown on the right. The width of the wound was measured manually (**P < 0.01; ***P < 0.001); the treatment group and the control group were compared by *t* test. **b** The expression of proteins important for migration and invasion were detected by western blot after TFP treatment in 4T1 cells. β-actin served as the loading control. **c** TFP inhibited the migration of 4T1 cell in the transwell migration assay. 4T1 cells were exposed to TFP for 18 h. The quantified values were shown in right (***P < 0.001). **d** TFP inhibited the invasion of 4T1 cell in the transwell invasion assay. 4T1 cells were exposed to TFP for 24 h. The quantified values were shown on the right (***P < 0.001). **e**, **f** TFP inhibits the growth of 4T1 brain metastasis in intracarotid model in vivo. The metastasis growth in the brain was monitored by noninvasive bioluminescence imaging technology (IVIS, PerkinElmer) in vivo every 3 days. The imaging was captured at the peak time after i.p. injection of 150 mg/kg d-luciferin. The imaging exposure time was 60 s. **f** The tumor burden was measured and judged by bioluminescence intensity (BLI). **g** TFP treatment prolongs the survival of mice bearing brain metastasis. The survival of the mice bearing brain metastasis was monitored everyday during the treatment (*P < 0.05). **h** Body weight changes of mice bearing brain metastasis during TFP treatment. The results showed that TFP treatment did not cause obvious weight changes compared with vehicle-treated mice

Metastasis of breast cancer, especially to brain, is the main cause of cancer-related death^5^. Migration and invasion are pivotal steps for the success of cancer metastasis^33^. Therefore, we assessed the effects of TFP on breast cancer cell migration and invasion. Firstly, we performed wound-healing assays using 4T1 and MDA-MB-231 cells. As shown in Fig. 6a and Supplementary Fig. 6A, the results indicated that the migration of TFP-treated 4T1 cells and MDA-MB-231 cells was significantly delayed as compared with vehicle-treated cells. The inhibition is time dependent in 4T1 cells. Transwell migration assay showed a similar result. Twenty hours of TFP treatment inhibited migration of 4T1 cells by 68% and 95.5% at 10 μM and 20 μM, respectively (Fig. 6c). Furthermore, we performed Matrigel invasion assay with transwell. The results showed that invasion of 10 μM TFP-treated cells was only 12.5% when compared with vehicle control cells (Fig. 6d). These findings suggested that TFP could inhibit migration and invasion of breast cancer cells. Moreover, we evaluated the expression of some proteins that have critical roles in tumor metastasis. The expression of both MMP-9 and p-FAK were decreased after treatment with TFP (Fig. 6b and Supplementary Fig. 6B). These observations indicated that TFP could inhibit breast cancer cells migration and invasion in vitro, suggesting its antimetastatic potential in vivo.

### TFP inhibited in vivo brain metastasis of TNBC cells

The in vivo anti-metastasis activity of TFP was further validated in breast cancer brain metastatic model. Luciferase-expressing 4T1 cells were injected into the right common carotid artery to establish the metastasis models. Bioluminescence imaging (BLI) technology was used to assess the tumor size in which the intensity of bioluminescent signal is positively related to the number of luciferase-expressing cells^34^. As shown in Fig. 6e, f, there is an obvious increase in brain luminescence starting day 7 after injection. On the basis of luminescence measuring, our data showed about 73.51% inhibition in brain metastasis growth by TFP treatment compared with luminescence signal in control group at day 10. Importantly, TFP treatment could prolong the survival of mice bearing metastatic TNBC tumor in the brain (Fig. 6g), while it did not cause obvious changes in the body weight (Fig. 6h). The median survival time of mice in TFP treatment group is 22 days compared with 19 days in control group. Similar results were observed with luciferase-expressing MDA-MB-231 cells (Supplementary Fig. 6C–E). These results suggested that TFP has the potential to inhibit the brain metastasis of TNBC in vivo.

## Discussion

TNBC is an aggressive breast cancer subtype that accounts for a disproportionate number of cancer-related deaths due to the absence of effective therapeutic strategies and agents^4,35^. TNBC patients are at high risk of metastasis to other organs and nearly 25–46% patients with TNBC are at a high risk of brain metastasis^36^. Therefore, finding new and effective treatment options for metastatic TNBC is more and more critical. Repurposed drug gives us the potential to identify effective, inexpensive anticancer agents.

Many clinical investigations have found an overall reduced risk of cancer in schizophrenic patients using neuroleptic drugs, suggesting antipsychotic drugs might have potential anticancer values for clinical treatment^18,37^. Indeed, limited publications have shown that some antipsychotic drugs such as penfluridol, chlorpromazine, and thioridazine displayed anticancer effects in different tumor models^37,38^. TFP is an approved antipsychotic drug for treating schizophrenia and shows good bioavailability in brain. Some studies have shown that TFP have anticancer effects on lung cancer and other cancer models^20,39–41^. However, there were few reports about its potential applications in treating metastatic TNBC, particularly brain metastases. Thus, our present study elucidated TFP’s in vitro and in vivo activity against metastatic TNBC as well as the possible underlying mechanisms.

TFP exhibited favorable antiproliferative activity in multiple cancer cells as shown by MTT assay. Next, we elucidated its inhibitory effect on TNBC and the underlying mechanisms using MDA-MB-231, MDA-MB-468, and 4T1 cells. The results showed that TFP has the potential to suppress TNBC via inducing G0/G1 cell cycle arrest and mitochondria-mediated apoptosis. Importantly, our work indicated that TFP could significantly suppress the growth of subcutaneous tumor and intracarotid brain metastasis tumor. Moreover, it prolonged the survival of mice bearing brain metastases of TNBC. TFP also showed good safety profiles during the treatment.

Abnormal cell cycle is a hallmark of cancer. Targeting this pathway has already shown a promise in treating breast cancer, and several drugs targeting cell cycle have been approved by FDA^42,43^. Recent studies in cell cycle indicated that the interaction among cyclins, CDKs and cyclin-dependent kinase inhibitors (CKIs) plays a fundamental role in cell cycle progression^13,44,45^. Cyclin D, cyclin E, and their specific interacted CDKs are the crucial regulators in G1 phase^21–23^. It had been recognized that cyclin D and cyclin E were oncogenic whose overexpression may be related with poor prognosis^23,46,47^. Cells will synthesize cyclin D in response to the mitogenic stimulation. Increased amount of cyclin D combines with CDK4 and CDK6 to phosphorylate the inhibitory protein retinoblastoma (Rb) in G1 phase, which leads to the dissociation of Rb from the transcription factor E2F and promote E2F-dependent transcription. E2F activation could promote a series of subsequent events that favor DNA replication and expression of cyclin E and CDK2. Upregulated cyclin E and CDK2 formed a positive feedback loop for the phosphorylation of Rb, which enhanced cells to cross the G1/S checkpoint^48,49^. CKIs are CDK inhibitors that possess the activity of tumor suppression. The most famous CKIs in G1 and S phases belong to p21 family that contains p21 and p27. They exert anticancer activities via inactivating CDK-cyclin complexes^49–51^. In our present study, the levels of cyclin D1 and cyclin E along with their specific interacting CDKs including CDK4 and CDK2 were downregulated in 4T1, MDA-MB-468, and MDA-MB-231 cells after TFP treatment. Moreover, the expression of p21 and p27 was upregulated in MDA-MB-231 and MDA-MB-468 cells.

Cell cycle arrest could lead to cell apoptosis^14,52^. In this study, we found TFP induced TNBC cell apoptosis. After treatment of TNBC cells with TFP, the level of the proapoptotic protein Bax was upregulated while the antiapoptotic protein Bcl-2 was downregulated. In addition, an increase expression of cleaved caspase-3 and a decrease in ΔΨm was observed. So we demonstrated that treatment with TFP could further activate the mitochondria-dependent intrinsic apoptotic pathway in TNBC cells.

The PI3K/Akt pathway is an important regulator of cell survival through multiple downstream targets and it has been shown that AKT could promote the activation of NF-kB by phosphorylation of IkB kinase (IKK), which in turn, augments the transcriptional activity of NF-kB p65/RelA, and promote cell survival^53^. In our present study, TFP decreased the expression of phosphorylated AKT, phosphorylated IkBa and reduced NF-kB p65 nuclear translocation. TFP might have inhibited TNBC cells survival via Akt/NF-kB signaling pathway.

We also tested TFP’s activity to inhibit subcutaneous tumors in BALB/c and NOD/SCID mice. We chose intraperitoneal injection instead of oral administration because the former administration route usually results in a higher drug concentration in the blood. Our goal in this study is to seek TFP’s potential for treating TNBC, and the possible dose and administration route in patient could be explored at a later stage. After an initial safety test in BALB/c mice, we chose 40 mg/kg as the dose in mice. TFP showed evidently inhibitory effect of 51.2% on 4T1 xenograft tumor growth in vivo when administered at 40 mg/kg, consistent with the in vitro findings. The tumor growth-suppressive effects of TFP were associated with reduced expression of Ki67 and increased expression of cleaved caspase-3 in tumor cells when compared with the vehicle group. Importantly, it showed good safety profiles during treatment. The mice did not show significant weight loss or other signs of toxicity as shown by H&E staining of major organs and hematological/serum biochemical parameters in 4T1 and MDA-MB-468 xenograft tumor models after treatment with TFP 40 mg/kg (Fig. 5e and Supplementary Fig. 5B).

TNBC are easily to metastasize to other important organs, and metastasis finally causes mortalities. Brain metastasis of TNBC lacks treatment strategies and drugs^5^. Due to its good penetration to brain, TFP’s ability to treat established brain metastasis was assessed in vivo by BLI technology in which the intensity of bioluminescent signal is positively related to the number of luciferase-expressing cells^33^. Our study showed that TFP treatment substantially suppressed the establishment of metastasized TNBC tumors in the brain of mice in the intracarotid model. And this suppression was more robust than the subcutaneous tumors, maybe due to the high penetration of TFP to the brain. More importantly, TFP treatment prolonged the survival of mice bearing brain metastasis.

In conclusion, our present study gives preliminary convincing results to convince TFP’s favorable antitumor and antimetastatic effects against TNBC in vitro and in vivo. However, the mechanisms by which TFP shows anticancer effects and its direct target are not fully understood, which prompted further investigation. To the best of our knowledge, there was no report about TFP’s potential applications in treating established TNBC brain metastases. Our work lays a foundation to repurpose TFP for treating TNBC, which currently lacks any effective treatment options. Therefore, our results suggested that TFP might be a potential therapeutic agent for TNBC growth and brain metastasis, which deserves further investigation.

## Materials and methods

### Materials

Trifluoperazine hydrochloride was purchased from AstaTech BioPharmaceutical Corp (Chengdu), and was dissolved in dimethyl sulfoxide (DMSO) for test in vitro. 3-(4,5-Dimethylthiazol-2-yl)-2,5-diphenyltetrazolium bromide (MTT), DMSO, propidium iodide (PI), Rhodamine-123 (Rh123), Hoechst 33342 were purchased from Sigma (St Louis, MO). Annexin V-FITC apoptosis detection kit was purchased from Roche (Indianapolis, IN). The primary antibodies were purchased from Cell Signaling Technology Company (Beverly, MA).

### Cell lines and cell culture

Human TNBC cells MDA-MB-468 were purchased from the Type Culture Collection of Chinese Academy of Science (Kunming, China). Other cells were purchased from ATCC (American Type Culture Collection). All of them were cultured in DMEM or DME/F-12 media containing 10% fetal bovine serum and 1% antibiotics (penicillin and streptomycin) under humidified condition with 5% CO_2_ at 37 °C.

### Cell viability assay

The effect of TFP on cell viability was assessed by the MTT assay. Briefly, cancer cells (2–8 × 10^3^ cells/well) were seeded in 96-well plates and incubated overnight. Then the cells were treated with different concentrations of TFP. After treatment for 24, 48 and 72 h, 20 μL of 5 mg/mL MTT solution was added to each well and incubated for an additional 2–4 h at 37 °C. The medium was subsequently removed and 150 μL DMSO was added to dissolve the purple-colored formazan crystal. Five minutes later, the absorbance was measured at 570 nm using Spectra MAX M5 microplate spectrophotometer (Molecular Devices, CA, USA) for living cells. All assays were replicated three times.

### The colony formation assay

TNBC cells (400–800 cells/well) were seeded in six-well plate and incubated overnight. Then the cells were treated with different concentrations of TFP for additional 7–14 days. The cells were washed with cold PBS before being fixed with 4% paraformaldehyde and stained with a 0.5% crystal violet solution for 15 min, and the colonies (>50 cells) were counted under a microscope. Data shown were the average of three independent experiments.

### Morphological analysis of cell nuclei

Cancer cells (1–2 × 10^5^ cells/well) were plated onto an 18-mm coverglass in six-well plate and incubated overnight. Then the cells were treated with different concentrations of TFP for 48 h. Finally the cells were stained by Hoechst 33342 (10 μg/mL) solution for 10 min in dark at room temperature followed by washing with cold PBS and fixed with 4% paraformaldehyde. Images were taken using a fluorescence microscope (Olympus, Tokyo, Japan).

### Cell cycle and apoptosis analysis by flow cytometry

The distribution of cell cycle was analyzed with PI staining by FCM. Briefly, breast cancer cells (8–20 × 10^4^ cells/well) were plated onto a six-well plate. After 24 h incubation, the cells were treated with different concentrations of TFP for 12 and 24 h. Then the cells were harvested and stained with PI followed by washing twice with cold PBS and fixed with 75% ethanol. Cell cycle distribution was measured by FCM. Data were analyzed with Novo Express 1.1.2 software. To confirm and quantify the apoptosis induced by TFP, we used the Annexin V-FITC apoptosis detection kit. The cells were harvested and stained with Annexin V-FITC for 5 min in dark at 4 °C and then stained with PI for 5 min, and finally detected by FCM. Data were analyzed by FlowJo software. Each experiment was replicated three times.

### Mitochondrial membrane potential (ΔΨm) assay

Mitochondrial membrane potential was measured by FCM. TNBC cells (8–20 × 10^4^ cells/well) were seeded in six-well plate. After incubation of 24 h, cells were treated with indicated concentrations of TFP for desired time. Then the cells were incubated with Rh123 (5 μg/mL) for 30 min in dark. The stained cells were then washed with cold PBS and detected by FCM.

### Measurement of ROS levels in cells

4T1, MDA-MB-231, and MDA-MB-468 cells were incubated with DCFH-DA (10 μM) at 37 °C for 30 min after exposure to various concentrations of TFP for the indicated time. Then the fluorescence intensity was tested by FCM.

### Immunofluorescence

The immunofluorescence assay was performed as previously described, with some modification^54^. In brief, tumor cells were plated onto a 14-mm coverglass in 24-well plate and incubated overnight. Different concentrations of TFP were added to treat cells for 48 h. Then cells were washed twice in PBS, fixed in 4% paraformaldehyde for 10 min and washed three times. Then unspecific binding sites were blocked with PBST containing 1% BSA and 0.05% Triton X-100. Subsequently, cells were incubated with primary antibody overnight at 4 °C. Goat anti-mouse secondary antibodies conjugated to FITC were used. Cell nuclei were stained with DAPI. Images were taken using a laser-scanning confocal microscopy (Nikon).

### Western blotting analysis

Cocktail was added into RIPA buffer at 1:1000 ratio before use. Cells were harvested and lysed in RIPA buffer (Beyotime, Beijing, China) on ice for 30 min after exposure to various concentrations of TFP for 72 h. Then, the cell lysate was centrifuged with 13,000 rpm at 4 °C for 15 min and then the supernatant was harvested. The concentration of protein was equalized by BCA method. Equal amount of total proteins was resolved by SDS-PAGE and transferred under the condition of 250 mA, 2 h onto the nitrocellulose membrane. After blocking with 5% fat-free milk soluble in TBS/T for 1.5 h at room temperature, the membranes were incubated with primary antibody overnight at 4 °C. The protein bands were visualized using an enhanced chemiluminescent substrate on horseradish peroxidase (Amersham, Piscataway, NJ) after incubating with horseradish peroxidase-conjugated secondary antibodies. The quantitation of the western blot results was based on three independent experiments using ImageJ.

### Wound-healing migration assay

4T1 cells (1 × 10^5^) and MDA-MB-231 cells (2 × 10^5^) were seeded in a 24-well plate. When cells grew to 80% confluence, we scraped the monolayer cells by 10 μL sterile tips and washed it with sterile PBS. Then fresh medium containing different concentrations of TFP was added. Cells were photographed after treatment with TFP for indicated time. Images were acquired using a microscope (Zeiss, Jena, Germany).

### Boyden chamber migration and invasion assay

Boyden chamber (8 μm pore size) migration assay was performed as previously described, with some modification^33,55^. 4T1 cells (4 × 10^4^) in 200 μL serum-free medium were added in the upper chamber, and 600 μL medium containing 10% FBS was added at the bottom. The same amount of 0.1% DMSO or TFP were added into both chambers and bottom. Cells were allowed to migrate for 18 h. Nonmigrated cells on the upper chamber were discarded using a cotton swab. The migrated cells were fixed with 4% paraformaldehyde and stained with 0.5% crystal violet solution for 15 min. Migrated cells were randomly selected fields, counted and photographed using a light microscope. Invasion assay was performed as described in previous studies^33,55^. Briefly, the upper surface of the transwell was coated with diluted Matrigel (1:4, 50 μL/well, BD Biosciences). When the diluted Matrigel was solidified, 4T1 cells (1.5 × 10^5^) in 200 μL serum-free medium were added in the upper chamber, and 600 μL medium containing 10% FBS was added at the bottom. Other steps were the same as above except the TFP treatment was 24 h.

### Subcutaneous xenograft and intracarotid brain metastasis models

Female BALB/c mice (6–8 weeks) were purchased from HFK Bioscience Co., Ltd. (Beijing, China). The experiments were approved and conducted in strict accordance with the regulations of the Animal Care and Use Committee of Sichuan University. Subcutaneous xenograft models were established as previously described^55^. Briefly, 100 μL media containing 2.5 × 10^5^ 4T1 cells and 5–10 × 10^6^ MDA-MB-468 cells were injected subcutaneously into the right flanks of female BALB/c mice and NOD/SCID mice, respectively. The mice were grouped into three groups (seven mice per group) at random when the tumor volume was about 100 mm^3^. Then the three groups of mice were treated with vehicle (v/v) (12.5% Cremophor El, 2.5% DMSO, 85% normal saline), 20 mg/kg TFP and 40 mg/kg TFP by intraperitoneal injection everyday, respectively. Tumor volume measured by a digital caliper and body weight of the mice were recorded every 3 days and clinical symptoms were observed everyday. The tumor volume was calculated according to the following formula: Tumor volume (mm^3^) = 0.52 × *L* × *W*^2^ where *L* is the length and *W* is the width.

Intracarotid brain metastasis models were established as described before^56^. Briefly, 4T1 and MDA-MB-231 cells expressing luciferase were harvested, washed and resuspended in sterile HBSS at a density of 50,000 cells/100 μL, and 100,000 cells/100 μL, respectively. Then the cells were injected into the right common carotid artery of BALB/c mice and BALB/c nude mice, respectively. Brain metastasis took place in all survived mice and the metastasis growth in the brain was monitored in vivo by noninvasive BLI technique (IVIS, PerkinElmer). Two days after injection, the mice were randomly separated into vehicle (six mice per group) and TFP treatment group (seven mice per group). The survival of the mice was also recorded.

### Immunohistochemistry

Briefly, the tumor sections of paraffin-embedded were stained with primary antibodies (P-AKT, Ki67 and cleaved caspase-3). Images were taken using a fluorescence microscope (Olympus, Tokyo, Japan).

### Toxicity evaluation

During the treatment of 4T1 xenograft on BALB/c mice and MDA-MB-468 xenograft on NOD/SCID mice, they were also observed everyday to investigate the side effects and toxicities after TFP treatment. On the 18th day, all animals were euthanized by extracting the eyeball blood. Blood was obtained for blood chemistry analysis and blood routine analysis. Histological examinations of heart, liver, spleen, lung, brain, and kidney were carried out after dissection by H&E staining.

### Statistical analysis

All the statistics were analyzed by Prism 6.0 software (GraphPad Software Inc.). Data were represented as means ± SD or SEM. Statistical significance was analyzed using Student’s *t* test and ANOVA and the considered statistical *P* values were labeled as follows: *P < 0.05; **P < 0.01; ***P < 0.001.

## Electronic supplementary material

Supplementary Figures

## References

[1] Ismail-Khan R, Minton S, Khakpour N (2015). Triple-negative breast cancer. Drugs.

[2] Siegel RL, Miller KD, Jemal A (2016). Cancer statistics 2016. CA Cancer J. Clin..

[3] Dietze EC, Sistrunk C, Miranda-Carboni G, O’Regan R, Seewaldt VL (2015). Triple-negative breast cancer in African-American women: disparities versus biology. Nat. Rev. Cancer.

[4] Tomao F (2015). Triple-negative breast cancer: new perspectives for targeted therapies. OncoTargets Ther..

[5] Ranjan A, Gupta P, Srivastava SK (2016). Penfluridol: an antipsychotic agent suppresses metastatic tumor growth in triple-negative breast cancer by inhibiting integrin signaling axis. Cancer Res..

[6] Bianchini G, Balko JM, Mayer IA, Sanders ME, Gianni L (2016). Triple-negative breast cancer: challenges and opportunities of a heterogeneous disease. Nat. Rev. Clin. Oncol..

[7] Mendes O, Kim HT, Lungu G, Stoica G (2007). MMP2 role in breast cancer brain metastasis development and its regulation by TIMP2 and ERK1/2. Clin. Exp. Metastas-..

[8] Kodack DP, Askoxylakis V, Ferraro GB, Fukumura D, Jain RK (2015). Emerging strategies for treating brain metastases from breast cancer. Cancer Cell..

[9] Aversa C (2014). Metastatic breast cancer subtypes and central nervous system metastases. Breast.

[10] Camidge DR (2010). Cell cycle-associated kinases as targets for therapy in lung cancer. J. Thorac. Oncol..

[11] Diaz-Moralli S, Tarrado-Castellarnau M, Miranda A, Cascante M (2013). Targeting cell cycle regulation in cancer therapy. Pharmacol. Ther..

[12] Hanahan D, Weinberg RA (2011). Hallmarks of cancer: the next generation. Cell.

[13] Vermeulen K, Van Bockstaele DR, Berneman ZN (2003). The cell cycle: a review of regulation, deregulation and therapeutic targets in cancer. Cell Proliferat.

[14] Xia Y (2014). SKLB316, a novel small-molecule inhibitor of cell-cycle progression, induces G2/M phase arrest and apoptosis in vitro and inhibits tumor growth in vivo. Cancer Lett..

[15] Ola MS, Nawaz M, Ahsan H (2011). Role of Bcl-2 family proteins and caspases in the regulation of apoptosis. Mol. Cell. Biochem..

[16] Ashkenazi A (2008). Targeting the extrinsic apoptosis pathway in cancer. Cytokine Growth Factor Rev..

[17] Gupta SC, Sung B, Prasad S, Webb LJ, Aggarwal BB (2013). Cancer drug discovery by repurposing: teaching new tricks to old dogs. Trends Pharmacol. Sci..

[18] Dalton SO (2006). Cancer risk among users of neuroleptic medication: a population-based cohort study. Br. J. Cancer.

[19] Ravn J (1987). Neuroleptic treatment and other factors modifying cancer risk in schizophrenic patients. Acta Psychiat Scand..

[20] Chen QY (2009). Molecular mechanism of trifluoperazine induces apoptosis in human A549 lung adenocarcinoma cell lines. Mol. Med. Rep..

[21] Fang F, Orend G, Watanabe N, Hunter T, Ruoslahti E (1996). Dependence of cyclin E-CDK2 kinase activity on cell anchorage. Science.

[22] Massagué J (2004). G1 cell-cycle control and cancer. Nature.

[23] Yu XJ (2012). Gambogenic acid induces G1 arrest via GSK3beta-dependent cyclin D1 degradation and triggers autophagy in lung cancer cells. Cancer Lett..

[24] Park SH (2015). Pharmacological activation of FOXO3 suppresses triple-negative breast cancer in vitro and in vivo. Oncotarget.

[25] Hengartner MO (2000). The biochemistry of apoptosis. Nature.

[26] Azad MB, Chen Y, Gibson SB (2009). Regulation of autophagy by reactive oxygen species (ROS): implications for cancer progression and treatment. Antioxid. Redox Signal..

[27] Martin KR, Barrett JC (2002). Reactive oxygen species as double-edged swords in cellular processes: low-dose cell signaling versus high-dose toxicity. Hum. Exp. Toxicol..

[28] Circu ML, Aw TY (2010). Reactive oxygen species, cellular redox systems, and apoptosis. Free Radic. Bio. Med..

[29] Simon HU, Hajyehia A, Levischaffer F (2000). Role of reactive oxygen species (ROS) in apoptosis induction. Apoptosis.

[30] Roy SK, Srivastava RK, Shankar S (2010). Inhibition of PI3K/AKT and MAPK/ERK pathways causes activation of FOXO transcription factor, leading to cell cycle arrest and apoptosis in pancreatic cancer. J. Mol. Signal..

[31] Chang F (2003). Involvement of PI3K/Akt pathway in cell cycle progression, apoptosis, and neoplastic transformation: a target for cancer chemotherapy. Leukemia.

[32] Vandermoere F, Yazidibelkoura IE, Adriaenssens E, Lemoine J, Hondermarck H (2005). The antiapoptotic effect of fibroblast growth factor-2 is mediated through nuclear factor-kappa B activation induced via interaction between Akt and I kappa B kinase-beta in breast cancer cells. Oncogene.

[33] Zhang T (2012). Cucurbitacin E inhibits breast tumor metastasis by suppressing cell migration and invasion. Breast Cancer Res. Treat..

[34] Dan MC, Xu T, Sayler GS, Ripp S (2010). In vivo bioluminescent imaging (BLI): noninvasive visualization and interrogation of biological processes in living animals. Sensors.

[35] Cleator S, Heller W, Coombes RC (2007). Triple-negative breast cancer: therapeutic options. Lancet Oncol..

[36] Lin NU (2013). Breast cancer brain metastases:new directions in systemic therapy. Ecancermedicalscience.

[37] Wu L (2014). Anti-tumor effects of penfluridol through dysregulation of cholesterol homeostasis. Asian Pac. J. Cancer Prev..

[38] Shin SY (2013). The antipsychotic agent chlorpromazine induces autophagic cell death by inhibiting the Akt/mTOR pathway in human U-87MG glioma cells. Carcinogenesis.

[39] Polischouk AG (2007). The antipsychotic drug trifluoperazine inhibits DNA repair and sensitizes non small cell lung carcinoma cells to DNA double-strand break induced cell death. Mol. Cancer Ther..

[40] Shin SY, Chang GK, Dong DH, Kim JH, Lee YH (2004). Implication of Egr-1 in trifluoperazine-induced growth inhibition in human U87MG glioma cells. Exp. Mol. Med..

[41] Shin SY, Choi BH, Kim JR, Kim JH, Lee YH (2006). Suppression of P-glycoprotein expression by antipsychotics trifluoperazine in adriamycin-resistant L1210 mouse leukemia cells. Eur. J. Pharm. Sci..

[42] Kim ES (2017). Abemaciclib: first global approval. Drugs.

[43] Kim ES, Scott LJ (2017). Palbociclib: a review in HR-positive, HER2-negative, advanced or metastatic breast cancer. Target Oncol..

[44] Sui L (2001). Implication of malignancy and prognosis of p27 (kip1), Cyclin E, and Cdk2 expression in epithelial ovarian tumors. Gynecol. Oncol..

[45] Delsal G, Loda M, Pagano M (1996). Cell cycle and cancer: critical events at the G1 restriction point. Crit. Rev. Oncog..

[46] Spruck CH, Won KA, Reed SI (1999). Deregulated cyclin E induces chromosome instability. Nature.

[47] Mishina T (2000). Cyclin E expression, a potential prognostic marker for non-small cell lung cancers. Clin. Cancer Res..

[48] Lapenna S, Giordano A (2009). Cell cycle kinases as therapeutic targets for cancer. Nat. Rev. Drug Discov..

[49] Schafer KA (1998). The cell cycle: a review. Vet. Pathol..

[50] Gudas JM (1999). Cyclin E2, a novel G1 cyclin that binds Cdk2 and is aberrantly expressed in human cancers. Mol. Cell. Biol..

[51] Burdon T, Smith A, Savatier P (2002). Signalling, cell cycle and pluripotency in embryonic stem cells. Trends Cell Biol..

[52] Zhu Y (2014). A novel small-molecule YLT205 induces apoptosis in human colorectal cells via mitochondrial apoptosis pathway in vitro and inhibits tumor growth in vivo. Cell. Physiol. Biochem..

[53] Jeong SJ, Pisemasison CA, Radonovich MF, Park HU, Brady JN (2005). Activated AKT regulates NF-kappa B activation, p53 inhibition and cell survival in HTLV-1-transformed cells. Oncogene.

[54] Tegeder I (2001). Inhibition of NF-kB and AP-1 activation by R- and S-flurbiprofen. Faseb J..

[55] Yang F (2015). Nifuroxazide induces apoptosis and impairs pulmonary metastasis in breast cancer model. Cell Death Dis..

[56] Zhang L (2015). Microenvironment-induced PTEN loss by exosomal microRNA primes brain metastasis outgrowth. Nature.

