# Supplementary material for: The antipsychotic agent trifluoperazine hydrochloride suppresses triple-negative breast cancer tumor growth and brain metastasis by inducing G0/G1 arrest and apoptosis
Source: Cell Death Dis. 2018 Sep 26;9(10):1006. doi: 10.1038/s41419-018-1046-3 (PMC6158270; doi:10.1038/s41419-018-1046-3)
Supplement: Supplementary file 1 — Supplementary Figures [file 41419_2018_1046_MOESM1_ESM.doc]

**Supplementary Figures**

**
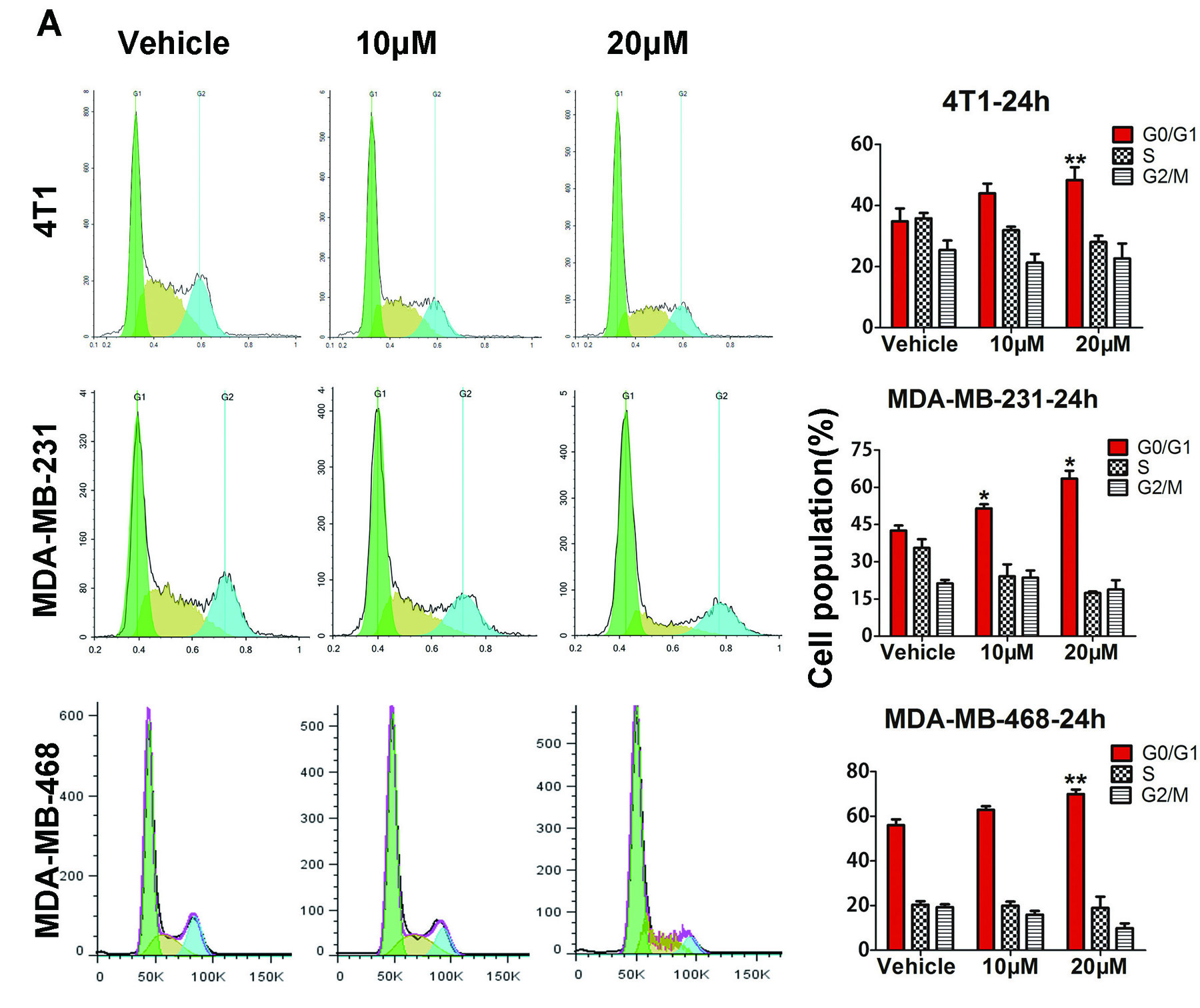
**

**Supplementary Figure 2. TFP induced G0/G1 arrest in TNBC cells.**

**(A)** 4T1, MDA-MB-231 and MDA-MB-468 cells were treated with indicated concentration of TFP for 24 hours, respectively. The distribution of cell cycle was analyzed by flow cytometry. Data shown were representative of 3 independent experiments. The quantification is shown in right panels. Data were expressed as mean ± S.D. from 3 experiments (**P<0.01; ***P<0.001), the treatment group and the control group were compared by t-test.


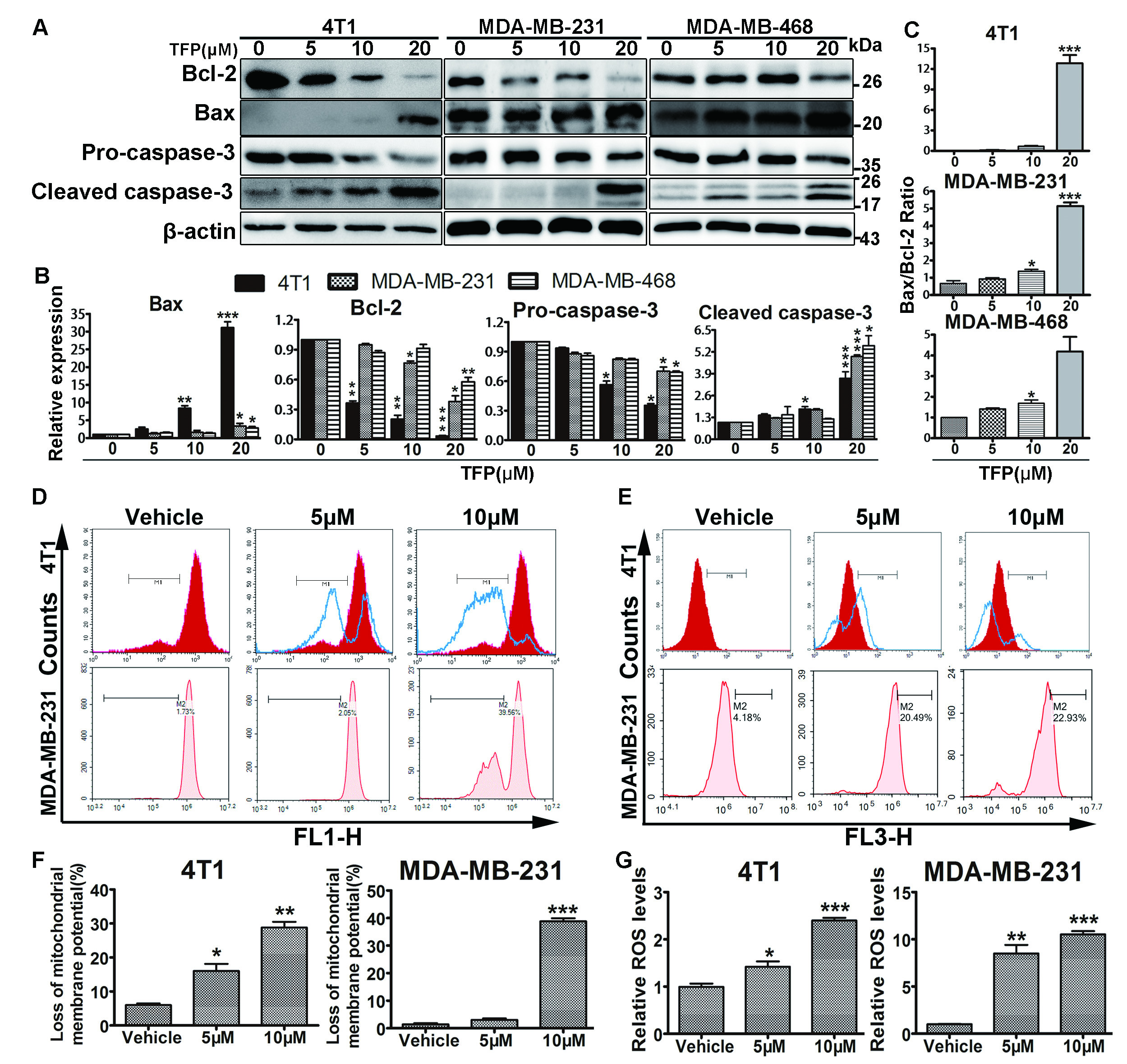


**Supplementary Figure 4. TFP induced mitochondria-mediated apoptosis of TNBC cells.**

**(A)** The expression of typical apoptosis-related proteins was determined by western blotting. 4T1, MDA-MB-231 and MDA-MB-468 cells were treated with TFP for 72 hours and the expression of Bcl-2, Bax, Pro-caspase-3 and Cleaved caspase-3 were detected. β-actin served as the internal control.

**(B)** Quantiﬁed results of the apoptosis related proteins expression after TFP treatment in 4T1, MDA-MB-231 and MDA-MB-468 cells (*P<0.05; **P<0.01; ***P<0.001), the treatment group and the control group were compared by t-test.

**(C)** The expression ratio of Bax/Bcl-2 was presented in the bar graphs (*P<0.05; ***P<0.001), the treatment group and the control group were compared by t-test.

**(D and F)** TFP treatment decreased mitochondrial membrane potential (ΔΨm) in 4T1 and MDA-MB-231 cells. Cells were treated with various concentrations of TFP for 24 hours and then stained with Rh123 to measure the change of ΔΨm by ﬂow cytometry. Quantiﬁed values were shown in (F) (*P<0.05; **P<0.01; ***P<0.001).

**(E and G)** TFP increased ROS levels in 4T1 and MDA-MB-231 cells. After treatment with various concentrations of TFP for 12 hours, 4T1 and MDA-MB-231 cells were incubated with DCFH-DA and then ROS levels were measured by DCF fluorescence with flow cytometry. Quantiﬁed values were shown in (G) (*P<0.05; **P<0.01; ***P<0.001).


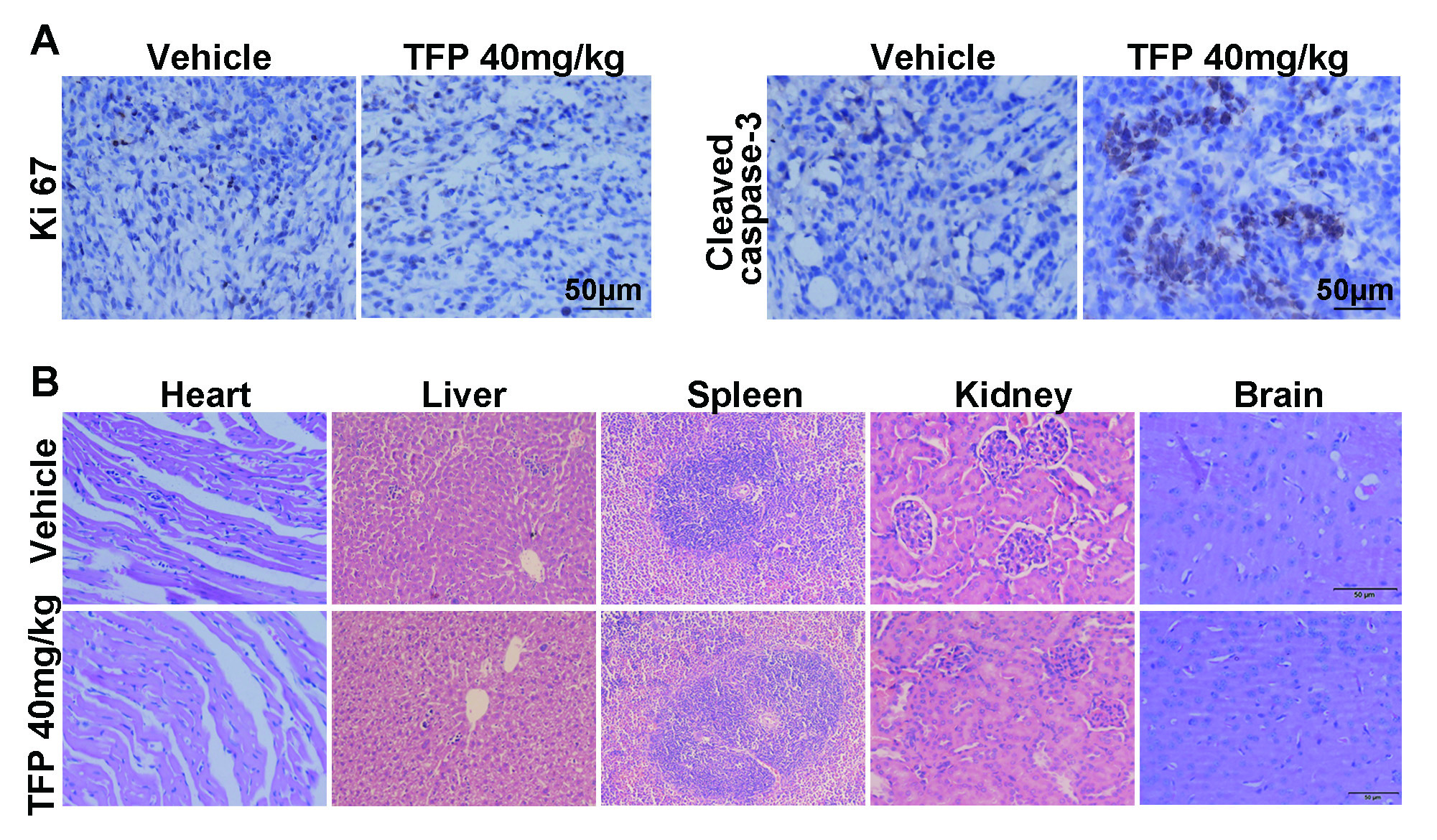


**Supplementary Figure 5.** **TFP’s inhibitory effects on the growth of TNBC in subcutaneous tumor model and safety profile.**

**(A)** Immunohistochemical analysis of tumor tissue was performed to measure the expressions of proliferation and apoptosis markers. The paraffin-embedded tumor tissues from the 4T1 tumor-bearing mice were stained with primary antibodies Ki67 and Cleaved caspase-3 to assess TFP’s effects on proliferation and apoptosis, respectively. Treatment with 40 mg/kg TFP caused a decreased staining of Ki67 and an increased staining of Cleaved caspase-3 in the tumor tissue. Scale bars represent 50 μm.

**(B)** TFP treatment didn’t cause obvious pathologic changes in major organs from the mice beard 4T1 xenograft tumor. Images shown are representatives from each group. Scale bars represent 50 μm.


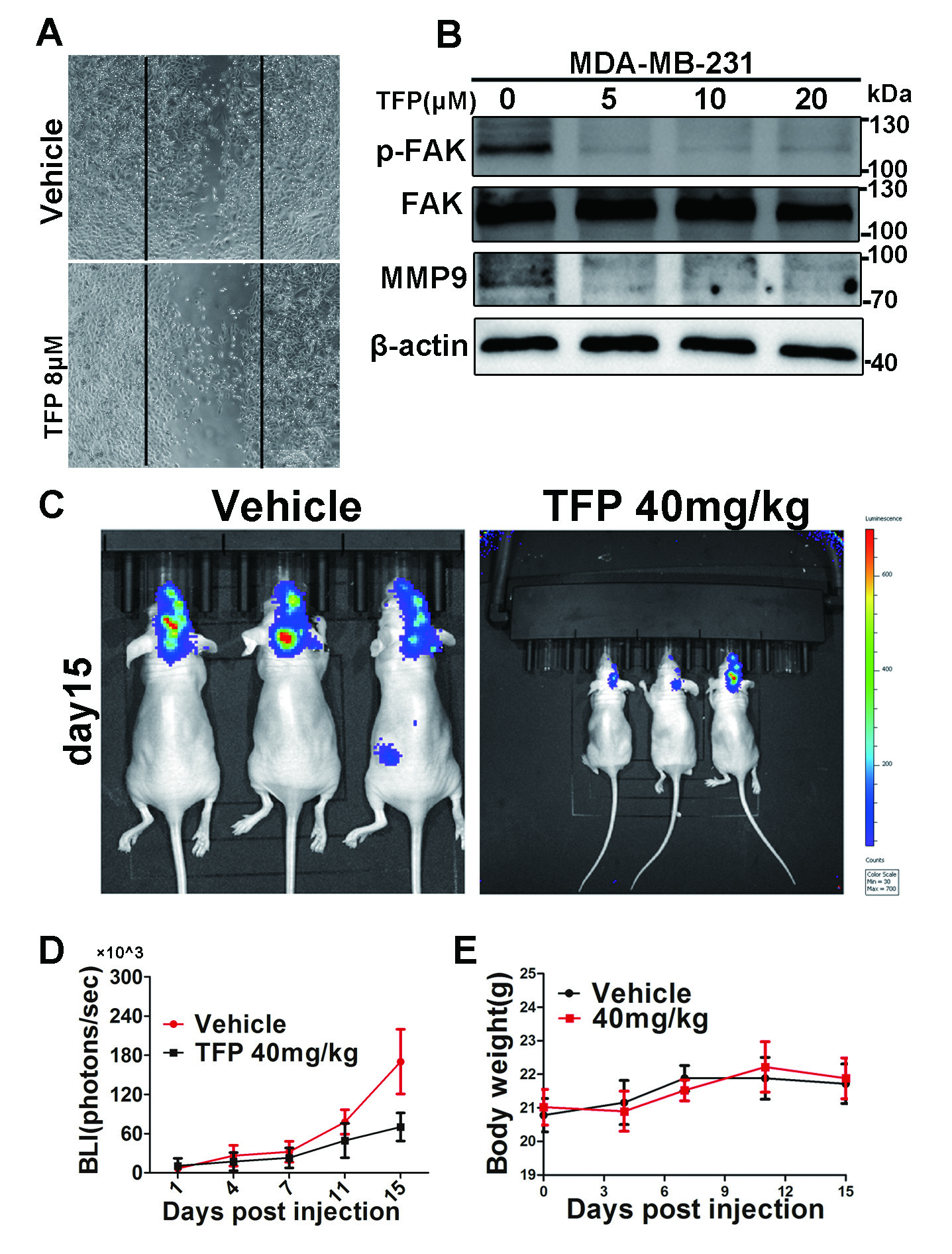


**Supplementary Figure 6. TFP’s inhibitory effects on brain metastasis of TNBC in intracarotid model.**

**(A)** TFP inhibited wound healing of MDA-MB-231 cells. MDA-MB-231 cells were exposed to TFP for 18 hours**.**

**(B)** The expression of proteins important for migration and invasion were detected by western blot after TFP treatment in MDA-MB-231 cells. β-actin served as the loading control.

**(C and D)** TFP inhibits the growth of MDA-MB-231 brain metastasis in intracarotid model *in vivo* (*P<0.05). MDA-MB-231 cells (100000 cells) expressing luciferase were injected into the right common carotid artery to establish brain metastasis model. The mice were treated with TFP or vehicle 2 days after the injection. The metastasis growth in the brain was monitored by *in vivo* by non-invasive bioluminescence imaging technology (IVIS, PerkinElmer) every 3 days. The imaging was captured at the peak time after i.p. injection of 150 mg/kg D-luciferin. The imaging exposure time was 60s. (D)The tumor burden was measured and judged by bioluminescence intensity (BLI).

**(E)** Body weight changes of bearing MDA-MB-231 brain metastasis mice during TFP treatment. The results showed that TFP treatment didn’t cause obvious weight changes compared with vehicle treated mice.
